# Enhanced glycolytic metabolism is associated with exhaustion and poor antitumor efficacy in a xenograft model of chimeric antigen receptor T cell therapy for sarcoma

**DOI:** 10.1186/2051-1426-1-S1-P21

**Published:** 2013-11-07

**Authors:** Adrienne H Long, Rimas J Orentas, Crystal L Mackall

**Affiliations:** 1 Pediatric Oncology Branch, National Institutes of Health, Bethesda, MD, USA; 2 Department of Microbiology-Immunology, Northwestern University, Chicago, IL, USA

## 

Chimeric antigen receptors (CARs) provide a promising new approach for generating T cell populations for the adoptive immunotherapy of cancer. CAR T cell (CART) therapies demonstrate activity against leukemias in preclinical and clinical studies, but CART targeting solid tumors have been less impressive. We hypothesized that the observed differences could be due to a more hostile microenvironment in solid tumors, and/or variable CAR potency. To address these issues, we created a CD19+GD2+ osteosarcoma (143B-CD19). This allowed us to compare the susceptibility of a solid tumor to both CD19-CART, which have potent anti-leukemic activity in vitro and in vivo, vs. GD2-CART, which show potent anti-tumor effects in vitro. ^51^Cr release demonstrated that CD19- and GD2-CART were equally active against 143B-CD19 in vitro. However, in vivo models showed a significant difference in anti-tumor efficacy. When NSG mice were injected with 143B-CD19 and treated with control, GD2- or CD19-CART 14d later, no difference in tumor growth was observed between GD2-CART and control treated animals. However, mice treated with CD19-CART showed complete eradication of their CD19+ disease. We also observed that CD19-CART expanded and persisted in vivo, whereas GD2-CART did not. Further comparison of GD2 vs. CD19 CARs demonstrated that GD2-CART depend more on glycolysis for metabolism, compared to CD19-CART or mock expanded controls. Using a Seahorse Extracellular Flux Analyzer, the ratio of the extracellular acidification rate (ECAR; measure of glycolysis) to oxygen consumption rate (OCR; measure of oxidative phosphorylation) of GD2-CART was found to be double that of CD19-CART or controls during in vitro expansion. This higher dependence on glycolytic metabolism was associated with a more exhausted phenotype, as GD2-CART expanded poorly, displayed higher rates of apoptosis, expressed higher levels of BLIMP-1, and expressed higher levels of exhaustion markers PD1 and TIM3 within 7 days of initial activation, compared to CD19-CART. GD2-CART also produced <10x lower levels of IL2, TNFα and IFNγ upon incubation with 143B-CD19, compared to CD19-CART. We conclude that the solid tumor microenvironment is not a barrier to effective CART therapy, but hypothesize that the enhanced glycolysis induced in GD2-CART leads to early exhaustion and diminished anti-tumor effects in vivo. Future work will seek to define the basis for the differential metabolism observed in CD19- vs. GD2-CART and will address the contribution of metabolism to T cell exhaustion.

